# Bridging the gap of knowledge and skills for diagnosis and treatment of painful neuropathy: Development and evaluation of pain education project for clinicians in primary care settings

**DOI:** 10.1097/MD.0000000000031606

**Published:** 2022-11-04

**Authors:** Rizaldy Taslim Pinzon, Vincent Ongko Wijaya, Esdras Ardi Pramudita, Theressia Handayani, Ranbebasa Bijak Buana

**Affiliations:** a Faculty of Medicine, Duta Wacana Christian University, Yogyakarta, Indonesia; b Bethesda Hospital, Yogyakarta, Indonesia; c Panti Rapih Hospital, Yogyakarta, Indonesia.

**Keywords:** neuropathy, pain education, primary care

## Abstract

The importance of pain education is widely accepted and recognized. This is a key part of educating the undergraduate and postgraduate healthcare workforce is an essential strategy for promoting effective pain practice. This study aims to evaluate the pain management module training courses for newly graduated doctors to address the knowledge gap between specialist care and primary care physicians. This was an observational study of an evaluation of a pain education project focused on neuropathic pain management core competency was provided. Multimodal teaching approaches such as didactic teaching and vignettes of cases discussion, video teaching, and learning module. A pretest survey was carried out to assess the baseline knowledge of the participants. Completion of the post-test and participant experience questionnaire were collected. Comparison of the pre-and post-test scores for all participants was undertaken using the Wilcoxon signed-ranked test with effect size calculated. The participant’s experience questionnaire scores were analyzed descriptively to produce mean and standard deviations from each question. A total of 274 participants completed all of the course sections from the average of 350 eligible participants. Of 274 participants, more than half were female (64.96%), with more than half participants being General Practitioner (54.38%) followed by a neurologist (35.04%). For all sessions, a Wilcoxon signed-rank test outlined that differences between all pre-and post-test scores were significant (*P* < .001). There was a marked improvement in the post-test as evidenced by statistically significant increases in mean scores differences. We developed an educational training courses for physicians to address the limitation in existing medical undergraduate training of neuropathic pain management. The training led to improvement in participant’s knowledge and skills with positive outcomes.

## 1. Introduction

Neuropathic pain is a chronic disabling condition with a high prevalence rate. It is an underdiagnosed and undertreated condition. The evaluation of neuropathy is challenging because of the variation of its etiologies, especially in local or primary care clinics with limited and lack of diagnostic workup. A previous national survey from the Indonesian Neurological Association showed that neuropathic pain was mostly associated with low back pain (28.6%), carpal tunnel syndrome (19.3%), and diabetic neuropathy (9.6%).^[1]^ A local survey from our hospital in Yogyakarta revealed that most geriatric patients with a complaint of neuromuscular pain in their visit had mixed type pain (86%) with neuropathic pain component.^[2]^ A study from a large tertiary hospital in Bandung reported that the prevalence of neuropathic pain was relatively high (31.6%).^[3]^ Preliminary studies from Medan and Bandung also showed that more than 50% of patients with type 2 diabetes had painful diabetic neuropathy or abnormalities on nerve conduction testing.^[3,4]^

In 2014, Indonesia launched a mandatory national health insurance system. In 2020, at least 80% of the Indonesian population had this insurance. Under this scheme, patients are free to access any primary care service. They also could obtain a referral letter from their general practitioner to access secondary care in referral hospitals.^[5]^ However, this referral system will cost more expenses by the government. Therefore, the primary care physicians have a crucial role as gatekeepers to secondary and tertiary care, and their role has been strengthened in the JKN implementation. There is very little data or research on neuropathic pain in local or primary care clinics. One major reason for the absence of this epidemiologic data on neuropathic pain was the lack of physician’s awareness or simple clinical instruments that can identify the characteristics of neuropathic pain.

Up to 40% of the primary care physicians in Indonesia have less than 5 years of experience in their current practice. Their undergraduate education in Schools of Medicine include little or no teaching of pain and pain management, coupled with minimum hours of pain training, results in their lack of confidence in assessing and diagnosing patients with pain, including neuropathic pain. Eventually, most of these students will work in the primary care clinics in their early days as freshly graduated doctors.

The training or workshop for diagnosing and managing neuropathies and other neurological conditions are often expensive, and only a few medical institutions offer proper training and workshops in pain management. In addition to limited resources, lack of knowledge and training for managing patients with neuropathic pain remains a significant obstacle to achieving adequate pain management for these patients, especially in primary care settings.

We believe that physicians, especially in low-resource settings such as primary care clinics, must have easily accessible and appropriate training for managing painful neuropathies. This study aims to address the gap between specialist care and primary care physicians through development of a pain management module and evaluation of training course for newly graduates doctors. We hypothesized that delivery of pain management modules and lectures may lead to an increase knowledge and skills in pain management.

## 2. Methods

### 2.1. Project development

This work was undertaken as part of grant funding awarded by the International Association for the Study of Pain (IASP). The grant call sought to support initiatives for improving pain education and practice in developing countries.

The project will be carried out through collaboration between Bethesda Hospital/ Duta Wacana Christian University School of Medicine with the Indonesian Medical Association Chapter Yogyakarta. We aimed to develop an on-line training module for Diagnosis and Treatment of Painful Neuropathy for Primary Care Physicians, Family Physicians, and Medical Interns. The module will contain theory, practice, therapeutic, and real case discussions with experts. A pre-post-test questionnaire will be used to assess the level of trainees’ performance at the beginning and the end of the training. The program will be carried out continuously with different topics and training sessions and collaborate with experts and participants in the Yogyakarta region.

### 2.2. Design

Overall, the project was carried out in a 4 months duration (2 months - module development including pre-recording video of the lecture/educational contents, 2 months-analysis of the results and report writing. From September to October, A team of experts led by a pain specialist and neurologist developed an online training module for this program. The project began with the team undertaking a review of materials to construct the curriculum module, including consultation from the Indonesian Pain Society. The project had a total of 10 face-to-face meetings on project coordination and module developments in Yogyakarta. The program was carried out online, and the major planning and coordination took place at Bethesda Hospital, Yogyakarta, Indonesia.

Participants were consecutively recruited and invited through medical institutions across Yogyakarta and other regions. Initially, this project targeted to recruit 250 to 300 physicians for this training. The online training sessions were carried out in October 2021 and divided into ten online training sessions with different topics for at least 1 session per week over 3 months. The project had a duration of 1-hour lecture (2 speakers and 1 moderator) and 30 minutes of discussion for each module session, including case and video studies. The facilitators and panelists were selected from well-known speakers (pain experts) from Indonesia. The organizers also collaborated with the other chapters of the Indonesian Pain Society and IASP members in the ASEAN region for the development of the modules.

These sessions were divided into 4 main sections, each section comprised of 2 to 3 modules (Table 1). These sessions were carried out on a web conference platform, and the module was accessible for all participants after the meeting. The project also recorded short pre-recording learning videos (15 minutes) for demonstration, which shown during module sessions, the videos topic including

**Table 1:** Overview of the curriculum of the online training.

Sections	Module title
Section 1: Understanding neuropathic pain	Module 1: The burden and challenges of pain management in indonesia
	Module 2: Classification and pathophysiology of neuropathic pain
Section 2: Mastering assessment and diagnosis	Module 3: Assessment of neuropathic pain in our daily practice
	Module 4: Challenges in diagnosis of neuropathic pain in limited resource settings
Section 3: Management approaches	Module 5: Understanding mechanism and evidence based pharmacological treatment for neuropathic pain
	Module 6: Non-pharmacological and simple interventional approaches in neuropathic pain management
	Module 7: Complementary and integrative therapy in neuropathic pain management
Section 4: Focus on specific conditions in our daily practice (with case discussion): Management at primary care level	Module 8: Painful Diabetic Peripheral Neuropathy and other peripheral neuropathies (including those from HIV and other infections), Cancer-related Neuropathic Pain Syndromes (case discussion with panelist)
	Module 9: Other Common Neuropathic Pain Conditions in Primary Care: Trigeminal Neuralgia, Low Back Pain with Neuropathic Component (HNP), Carpal Tunnel Syndrome, Post Herpetic Neuralgia
	Module 10: Central neuropathic pain (Central Post-Stroke Pain, Post-Spinal Cord Injury Pain, Multiple Sclerosis)

1. How to diagnose (history taking & physical examination);
2. The use and application of Diabetic Neuropathy Symptom and Diabetic Neuropathy Examination scores;
3. Practical Approach to different etiologies of Neuropathic Pain;
4. Introduction and Use of Monofilament to Diagnose Peripheral Neuropathy.

These videos were presented during module 3 and 4 in Assessment and Diagnosis Section (Section 2).

Prior to accessing each training section, each participant was required to complete a pretest survey to assess their baseline knowledge of the related modules. Completion of the post-test and participant experience questionnaire after each section was required for accessing/obtaining a certificate of completion. In total, there were 6 tests comprised of 3 pretests and 3 post-tests. These tests were carried out for Sections 1 and 2 together, Section 3 and Section 4. Pretests were carried out at the beginning of module 1, module 5 and module 8 and the post-tests at the end of module 4, module 7, and module 10.

Participants who joined all module sessions would receive a total of 3 certificates. The certificate of completion was accredited and issued by the Indonesian Medical Association, and the participant was eligible for CPD points.

### 2.3. Outcomes

This study aims to evaluate the online pain management module training courses for newly graduated doctors to address the knowledge gap between specialist care and primary care physicians. The participant’s knowledge will be assessed by comparing the pre- and post-test scores for all participants—the pre- and post-test comprises of multiple-choice questions with a single-correct option. The post-test replicated the same items as those presented during the pretest, and both were used to assess changes in pain knowledge.

Prior to accessing the modules, each student was required to complete a pretest survey to assess their baseline knowledge of pain and its management. The pretest comprised of 5 single-correct option, multiple choice questions, each allotted a mark of 20 points with no total benchmark score. Modules were then completed sequentially through the module numbers. At the end of each section, students were required to complete an end-of-module assessment comprising of 5 multiple-choice based on content from the module, with 5 single-correct options and multiple choice to qualify for progression to the next module.

At the end of module 10, The participant experience questionnaire contains ten questions and is designed to evaluate the participants’ experience of the online module. Each question is scaled from 0 (not satisfied) to 10 (very satisfied). The questions are linked to the participant’s interest, expectation, attractiveness, and novelty of the module. These responses will be recorded to update and improve the training modules.

### 2.4. List of measurable outcomes

- Participants attendance and completion of the modules.
- Pre-and post-test scores.
- Participant experience questionnaire scores.

### 2.5. Statistical analysis

Data analysis included pretest and post-test scores. To describe the baseline characteristics of participant’s, relevant descriptive statistics (frequencies, means and standard deviations as appropriate) were calculated. The normality of distribution of the data was assessed using the Shapiro–Wilk test. Comparison of the pre-and post-test scores for all participants was undertaken using the Wilcoxon signed-ranked test with effect size calculated. The participants experience questionnaire scores was analyzed descriptively to produced mean and standard deviations from each questions. Missing data was excluded from the analysis.

### 2.6. Ethical consideration

Informed consent was obtained from each participant. The investigators obtained ethical clearance from the Bethesda Hospital Research Ethics Committee by the number of 101/D.01/FK/2021.

## 3. Results

The average course attendance for 4 days of the training course was 350 participants. A total of 274 participants completed all of the course sections from the average of 350 eligible participants. Of 274 participants, more than half were female (64.96%), with more than half participants being General Practitioner (54.38%) followed by a neurologist (35.04%) (see Table 2).

**Table 2:** Participant’s profiles.

Participant’s profiles	N (274)	%
**Gender**		
Male	96	35.04
Female	178	64.96
**Specialization**		
General practitioner	149	54.38
Neurologist	96	35.04
Other specialists	13	4.74
Others	16	5.84

### 3.1. Pre- and post-test scores and level of change

The modules scores for both pre-and post-test were found to be normally distributed. The mean pretest score of the participants on day 1 was 77.03 (SD = 22.2), increasing to 88.23 (SD = 18.4) on the post-test. On day 2, the mean pretest score of the participants was 74.68 (SD = 21.07), increasing to 87.64 (SD = 17.17). On day 3, the mean pretest score of the participants was 75.38 (SD = 19.93), increasing to 85.13 (SD = 17.66) following the completion of the last modules. For all sessions, a Wilcoxon signed-rank test outlined that differences between all pre-and post-test scores were significant (*P* < .001). There was a marked improvement in the post-test as evidenced by statistically significant increases in mean scores differences. In addition, the highest post-test scores across all sessions occurred on day 1, while the lowest post-test scores were seen on day 3 (Table 3).

**Table 3:** The effect of pain module & training on participant’s knowledge.

Variables	n	Mean (SD)	Median (IQR)	Z-stat	NR PR Ties	*P* value
Pre-test Day 1	243	77.03 (22.2)	80 (100)	−7.924*	19 111 113	<.001
Post-test Day 1	243	88.23 (18.4)	100 (80)			
Pre-test Day 2	233	74.68 (21.07)	80 (100)	−8.064*	19 113 101	<.001
Post-test Day 2	233	87.64 (17.17)	100 (80)			
Pre-test Day 3	195	75.38 (19.93)	80 (100)	−6.269*	17 81 97	<.001
Post-test Day 3	195	85.13 (17.66)	100 (80)			

NR *=* negative ranks, PR *=* positive ranks, Z = Wilcoxon Signed-ranked test.

* *P* < .001.

**Figure 1.**
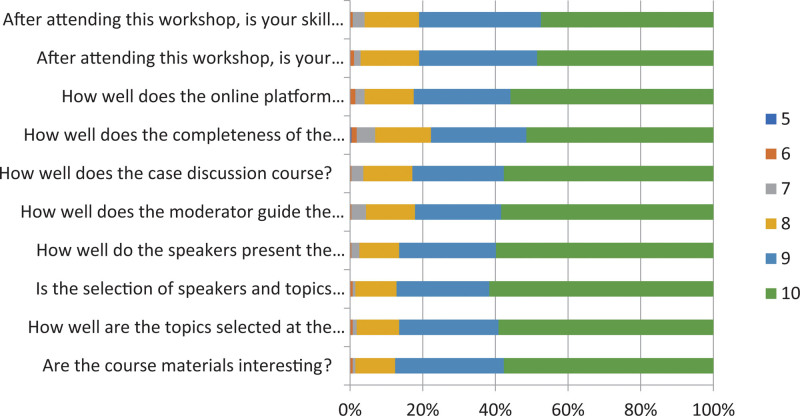
Distribution of participants’ experience scores towards the training module.

Participants’ experience data was obtained from all 274 participants who completed the training course. Overall, participants’ experiences were positive (see Table 4). The highest-rated experience item was “speakers selection” with a mean score of 9.47 (SD = 0.78), “how well the speakers presented the course,” “how interesting the course materials are,” and “how well the topic selection” were tied for the second-highest-rated experience item (mean = 9.43). The lowest rated experience item was the completeness of the modules, although it was still relatively high (mean = 9.2). The distribution of participants’ experience scores for the training module is shown in Figure 1.

**Table 4:** Mean scores of participants’ experience towards the training module.

	Questions	Mean (SD)
1	Are the course materials interesting?	9.43 (0.77)
2	How well are the topics selected at the event?	9.43 (0.79)
3	Is the selection of speakers and topics presented appropriately?	9.47 (0.78)
4	How well do the speakers present the materials?	9.43 (0.80)
5	How well does the moderator guide the workshop?	9.36 (0.89)
6	How well does the case discussion course?	9.37 (0.86)
7	How well does the completeness of the modules?	9.2 (1.01)
8	How well does the online platform performance in the course?	9.33 (0.91)
9	After attending this workshop, is your knowledge in diagnosing and managing neuropathy increased?	9.26 (0.87)
10	After attending this workshop, is your skill in diagnosing and managing neuropathy increased?	9.24 (0.88)

Likert Scale, 1-10. Value index for awareness-related items: Disagree strongly = 0, Agree strongly = 10.

## 4. Discussion

IASP defined *neuropathic pain* as “pain caused by a lesion or disease of the somatosensory nervous system.” The prevalence of neuropathic pain in the general population is estimated between 7% to 8% and 20% to 25% in individuals with chronic pain.^[6]^ National prospective survey in Indonesia reported that from 8000 participants with pain complaints, 21% had at least 1 symptom of neuropathic pain.^[1]^

Neuropathic pain has a diverse and large variety of underlying disease or lesion origins that are usually difficult to treat, such as trauma to the nervous system, vascular disease, nerve compression, infection disease, hereditary syndromes, metabolic disease, or neurological disease.^[7]^ Because of its complexity, neuropathic pain is often challenging to diagnose and treat, thus leading to underdiagnosis and ineffective treatment by physicians.

Over the decade, the interest in neuropathic pain studies is steadily increased, it indicated by the increasing number of research studies devoted to neuropathic pain, as shown in the PubMed index. The research publication increased from 915 in 2010 to 2767 in 2020 and 2985 in 2020. The expansion of neuropathic pain knowledge may lead to revisions of the definition, the diagnosis, and treatment of neuropathic pain. The complexity of neuropathic pain and constant revision on definition, diagnosis, and treatment may lead to challenges such as information dissemination and alignment of diagnosis and treatment for physicians, especially in primary care.

All Indonesian citizens are obliged to have the government’s national health insurance. The national health insurance policy state that the patient is obliged to receive healthcare in primary care before being referred to specialist care. Suppose the diagnosis and treatment of painful neuropathy can be managed early and appropriately in primary care. In that case, this can reduce the referral rate for specialist care in secondary and tertiary hospitals, thus reducing the total cost and time of treatment while improving the level of primary care physicians’ skill, thus reducing healthcare costs and helping the country’s economy in the long term. As the world’s largest island country, Indonesia is challenged in information dissemination and neuropathic pain diagnosis and treatment alignment. The problem such as distance between the islands, cost of transportation, and distribution of pain experts are examples of a problem that needs to be solved. An online training course is one of the best options to solve this problem.

The online training course offers multiple benefits for healthcare professionals in developing countries, including flexible learning, time efficiency, reduced costs of transportation and materials, easy accessibility and updated content, and the ability to accommodate teaching at a distance. Several studies reported positive results of online learning in developing countries. A recent study about online learning effectiveness in Indonesia reported improved concentration, understanding, and student motivation to learn.^[8]^ Another study highlighted the participant’s knowledge improvement after online learning.^[9]^ In line with previous studies, our results also show improved participant knowledge about neuropathic pain after participating in the online pain management training courses. These results show the promising potential of the online course in Indonesia, especially for improving neuropathic pain information dissemination and alignment of neuropathic pain diagnosis and treatment. Online training may also reshape the quality and curriculum of healthcare education in Indonesia. By improving and utilizing this approach to deliver neuropathic pain education in Indonesia, the health care system may also improve and eventually reduce the disease burden for the country.

Although the online course has significant potential, it has implementation challenges. A literature review on online learning in developing countries suggested that the lack of technological and financial infrastructure, such as inadequate internet access and stability have become an apparent problem in online learning applications.^[10]^ another study in India also reported that more than half of the participants are concerned about cyber security and an unstable internet connection as the major constrain for online learning.^[11]^

If the results of this program are positive, we plan to implement this online module training throughout other regions in Indonesia. Therefore, this program has the potential to change the fundamental quality of health education in the whole country.

## 5. Conclusion

We developed an educational training courses for physicians to address the limitation in existing medical undergraduate training of neuropathic pain management. The training led to improvement in participant’s knowledge and skills with positive outcomes. Future implementation and utilization of these training platform has the potential to improve pain education and management in Indonesia.

## Acknowledgments

The authors would like to thank all the instructors involved for their various role in the development of this program: Andi Muhammad Takdir Musba; Dessy Rakhmawati Emril; Yudiyanta; I Putu Eka Widyadharma; Yusak Siahaan; Jimmy Barus.

## Author contributions

**Conceptualization:** Rizaldy Taslim Pinzon, Vincent Ongko Wijaya, Esdras Ardi Pramudita.

**Data curation:** Vincent Ongko Wijaya, Ranbebasa Bijak Buana.

**Formal analysis:** Vincent Ongko Wijaya.

**Funding acquisition:** Rizaldy Taslim Pinzon, Theressia Handayani.

**Investigation:** Esdras Ardi Pramudita, Theressia Handayani, Ranbebasa Bijak Buana.

**Methodology:** Vincent Ongko Wijaya, Esdras Ardi Pramudita, Theressia Handayani, Ranbebasa Bijak Buana.

**Project administration:** Rizaldy Taslim Pinzon, Esdras Ardi Pramudita, Theressia Handayani.

**Resources:** Rizaldy Taslim Pinzon, Theressia Handayani.

**Software:** Ranbebasa Bijak Buana.

**Supervision:** Rizaldy Taslim Pinzon, Esdras Ardi Pramudita, Theressia Handayani.

**Validation:** Rizaldy Taslim Pinzon, Esdras Ardi Pramudita.

**Visualization:** Ranbebasa Bijak Buana.

**Writing – original draft:** Vincent Ongko Wijaya, Esdras Ardi Pramudita, Theressia Handayani, Ranbebasa Bijak Buana.

**Writing – review & editing:** Rizaldy Taslim Pinzon, Vincent Ongko Wijaya, Ranbebasa Bijak Buana.
